# A Novel, Orally Bioavailable, Small-Molecule Inhibitor of PCSK9 With Significant Cholesterol-Lowering Properties In Vivo

**DOI:** 10.1016/j.jlr.2022.100293

**Published:** 2022-10-06

**Authors:** Alexandra K. Suchowerska, Geurt Stokman, James T. Palmer, Phillip A. Coghlan, Elsbet J. Pieterman, Nanda Keijzer, Gilles Lambert, Kevin Chemello, Ali K. Jaafar, Jasneet Parmar, Liping Yan, Yingtao Tong, Lin Mu, Hans M.G. Princen, James Bonnar, Benny J. Evison

**Affiliations:** 1 Nyrada Inc., Gordon, New South Wales, Australia; 2 Metabolic Health Research, The Netherlands Organization of Applied Scientific Research, Leiden, The Netherlands; 3 Pharmaceutical Discovery Consultation, Warrandyte, Victoria, Australia; 4 i-Chem Consulting, Condell Park, New South Wales, Australia; 5 Laboratoire Inserm UMR 1188 DeTROI, Universite de la Reunion Plateforme CYROI, Sainte Clotilde, France; 6 DMPK/Exploratory Toxicology Department, Shanghai ChemPartner Co., Ltd., Shanghai, China

**Keywords:** PCSK9, cardiovascular disease, hypercholesterolemia, small-molecule, lipoproteins, apolipoproteins, drug therapy, LDL, statins, lysosomal degradation, ALT, Alanine transaminase, AST, Aspartate transaminase, CETP, Cholesteryl Ester Transfer Protein, CHOD-PAP, cholesterol oxidase phenol 4-aminoantipyrine peroxidase, CRISPR-Cas9, clustered regularly interspaced short palindromic repeats - CRISPR-associated protein 9, FPLC, Fast protein liquid chromatography, LDLR, Low density lipoprotein receptor, mRNA, messenger ribonucleic acid, PBMC, peripheral blood mononuclear cells, PCSK9, Proprotein convertase subtilisin kexin type 9, SREBF-2, Sterol regulatory element binding transcription factor 2, WTD, Western Type Diet

## Abstract

Proprotein convertase subtilisin kexin type 9 (PCSK9) inhibits the clearance of low-density lipoprotein (LDL) cholesterol (LDL-C) from plasma by directly binding with the LDL receptor (LDLR) and sending the receptor for lysosomal degradation. As the interaction promotes elevated plasma LDL-C levels, and therefore a predisposition to cardiovascular disease, PCSK9 has attracted intense interest as a therapeutic target. Despite this interest, an orally bioavailable small-molecule inhibitor of PCSK9 with extensive lipid-lowering activity is yet to enter the clinic. We report herein the discovery of NYX-PCSK9i, an orally bioavailable small-molecule inhibitor of PCSK9 with significant cholesterol-lowering activity in hyperlipidemic APOE∗3-Leiden.CETP mice. NYX-PCSK9i emerged from a medicinal chemistry campaign demonstrating potent disruption of the PCSK9-LDLR interaction in vitro and functional protection of the LDLR of human lymphocytes from PCSK9-directed degradation ex vivo. APOE∗3-Leiden.CETP mice orally treated with NYX-PCSK9i demonstrated a dose-dependent decrease in plasma total cholesterol of up to 57%, while its combination with atorvastatin additively suppressed plasma total cholesterol levels. Importantly, the majority of cholesterol lowering by NYX-PCSK9i was in non-HDL fractions. A concomitant increase in total plasma PCSK9 levels and significant increase in hepatic LDLR protein expression strongly indicated on-target function by NYX-PCSK9i. Determinations of hepatic lipid and fecal cholesterol content demonstrated depletion of liver cholesteryl esters and promotion of fecal cholesterol elimination with NYX-PCSK9i treatment. All measured in vivo biomarkers of health indicate that NYX-PCSK9i has a good safety profile. NYX-PCSK9i is a potential new therapy for hypercholesterolemia with the capacity to further enhance the lipid-lowering activities of statins.

Elevated plasma low-density lipoprotein (LDL) cholesterol (LDL-C) levels have long been linked with cardiovascular disease (CVD) (1, 2), a leading cause of death in humans worldwide (3). Removal of LDL-C from plasma is predominantly achieved through the action of the LDL receptor (LDLR), a transmembrane protein expressed at the plasma membrane of liver cells and responsible for the internalization of LDL particles via receptor-mediated endocytosis (4, 5). Proprotein convertase subtilisin kexin type 9 (PCSK9) is a liver-derived plasma serine protease that binds to the epidermal growth factor-like repeat A domain of LDLR, causing cellular internalization of the receptor (6). The PCSK9–LDLR complex subsequently translocates to the endosome–lysosomal compartment, where LDLR is degraded, resulting in a net decrease in the clearance of plasma LDL-C and elevated LDL-C levels (7, 8). Through this mechanism, PCSK9 regulates plasma LDL-C levels by modulating the degree of LDL particle clearance via LDLR-mediated hepatic uptake (9).

In 2003, the identification of gain-of-function mutations in the *PCSK9* gene confirmed its importance in cholesterol metabolism, with carriers of the mutations demonstrating elevated levels of LDL-C concomitant with increased CVD risk (10). PCSK9 has since been clinically validated as a therapeutic target for the treatment of hypercholesterolemia and atherosclerosis (11). The clinical introduction of anti-PCSK9 monoclonal antibodies alirocumab (12) and evolocumab (13) has been successful in lowering plasma LDL-C levels by 50%–70% and reducing cardiovascular events in patients (13, 14, 15). While these antibodies provide a viable treatment option for hypercholesterolemia, their use is tempered by clear disadvantages. For many, these monoclonal antibodies are prohibitively expensive (16), and there is a requirement for subcutaneous injections every 2 to 4 weeks (12, 13), both of which have restricted their widespread use (16).

A range of different strategies to inhibit or ablate PCSK9 function are at various stages of development. Strategies that have been pursued include the application of small interfering RNAs (17), CRISPR-Cas9–based technologies (18, 19), an anti-PCSK9 vaccine (20), anti-secretagogues (21, 22), peptide macromolecules (23), and orally bioavailable antisense oligonucleotides (24). Despite progress in targeting PCSK9 as a therapeutic modality, an orally bioavailable small-molecule inhibitor of PCSK9 is yet to enter the clinic (16). The absence of a small-molecule clinical candidate likely reflects the challenging nature of disrupting the PCSK9-LDLR interaction with these agents. The epidermal growth factor-like repeat A–interacting site of PCSK9 is ∼ 500 Å^2^ in surface area and is rather flat and featureless, bereft of any apparent pockets, grooves, or crevices appropriate for binding by small molecules (25, 26, 27). Nevertheless, a small-molecule inhibitor of PCSK9 is highly valued as a treatment option for hypercholesterolemia given its capacity to provide a cost-competitive alternative to anti-PCSK9 monoclonal antibodies, coupled with the potential for ease of oral administration (16).

Despite the challenging nature of its discovery, we recently reported the identification and characterization of compound 3f as a small-molecule inhibitor of PCSK9 (28). In a proof-of-concept study, compound 3f demonstrated lipid-lowering activity in decreasing the plasma cholesterol levels of wildtype mice by ∼10% following subcutaneous injection (28). It was also established early on that compound 3f was poorly orally bioavailable (*F* = 0.527%), a property that necessitated subcutaneous injection of the compound to achieve this modest reduction in plasma cholesterol (28). A medicinal chemistry campaign was launched to identify an orally bioavailable analog of compound 3f with superior bioactivity in lowering plasma LDL-C levels. We report herein the discovery of NYX-PCSK9i, a new orally bioavailable analog of compound 3f that disrupted the PCSK9-LDLR interaction at submicromolar levels in vitro and significantly reduced plasma cholesterol in hyperlipidemic APOE∗3-Leiden.CETP mice (29, 30, 31, 32). In addition, NYX-PCSK9i enhanced plasma cholesterol lowering by atorvastatin, with the combination generating an additive effect in this murine model of hyperlipidemia. To our knowledge, this is the first study showing that a combination treatment consisting of a small-molecule inhibitor of PCSK9 and a statin is highly effective in lowering LDL-C in vivo.

## Materials and Methods

### Materials

DMSO, BSA, PEG 6000, glycine, mouse anti-α-tubulin antibody, and Ficoll Paque Plus were purchased from Sigma (St Louis, MO and Saint Quentin-Fallavier, France). Alirocumab was obtained from Sanofi (Chilly-Mazarin, France), while atorvastatin was provided by the Leiden University Medical Center Pharmacy (Leiden, The Netherlands). Semi-synthetic mouse chow mimicking a Western-type diet (WTD) [containing 15% saturated fat and 0.15% cholesterol (w/w)]) was from Ssniff Spezialdiäten GmbH (Soest, Germany). Solutol HS15 was from BASF (Lugwigshafen, Germany) and Merck Life Sciences (Amsterdam, The Netherlands), while CB 300 K2E microvettes were obtained from Sarstedt (Nürnbrecht, Germany). RPMI and FBS were from Life Technologies (Saint Aubin, France), while Mouse PCSK9 Quantikine ELISA kits, goat anti-mouse-LDLR antibody, an allophycocyanin-conjugated antibody against human LDLR, and an IgG1 isotype control antibody were acquired from R&D Systems (Lille, France). The HRP-conjugated rabbit anti-goat-IgG antibody was obtained from Bio-Rad (Hercules, CA) while an HRP-labeled horse anti-mouse-IgG antibody was purchased from Cell Signaling Technology (Beverley, MA). For gel filtration, Superose® 6 PC 3.2/30 columns were purchased from GE Healthcare (Chicago, IL). CircuLex PCSK9-LDLR in vitro binding assay kits and recombinant mutant PCSK9-D374Y were obtained from CycLex (Nagano, Japan). Cholesterol CHOD-PAP kits were from Roche (Mannheim, Germany). The RNA extraction kit STAT-60 was purchased from AMSBio (Abingdon, UK), while a high-capacity RNA-to-cDNA kit and a TaqMan® Universal PCR Master Mix were acquired from Applied Biosystems (Waltham, MA). All quantitative polymerase chain reaction primers were from the TaqMan® Assay service (Applied Biosystems).

### Methods

#### Chemical synthesis

NYX-PCSK9i (IUPAC name (3-{[(S)-3-Amino-1-piperidyl]methyl-5-(4-methyl-1H imidazole-1-yl)phenylamino)(4-phenyl-2-pyridyl)formaldehyde) was synthesized at Jubilant Biosys Limited (Noida, India) and Shanghai ChemPartner Co. Ltd. (Shanghai, China). Each batch was prepared as the free base at a purity of greater than 95%. Representative chemical characterization data (^1^H-NMR and LCMS spectra) are provided in the Supplementary Information.

#### In vitro PCSK9-LDLR binding and ex vivo lymphocyte LDLR surface expression assays

NYX-PCSK9i was screened for its inhibitory activity against the PCSK9-LDLR interaction using a CircuLex PCSK9 in vitro binding assay kit (Cat# CY8150) according to the manufacturer’s instructions and as previously described (28). The inhibitory activity of NYX-PCSK9i was then tested in primary human lymphocytes, both with and without recombinant PCSK9-D374Y as previously detailed (28). Ethics was granted by the Comité de Protection des Personnes Sud Méditerranée (ID: 2020-A00196-33). Studies using human samples abided by the Declaration of Helsinki principles.

#### Pharmacokinetic studies

Pharmacokinetic study protocols were reviewed and approved by the Institutional Animal Care and Use Committee of ChemPartner (Shanghai, China) under ethics A998HL0119. Female C57BL/6 mice aged 10–14 weeks (18–21 g) were purchased from Jihui Laboratory Animal Co. Ltd. (Shanghai, China) and were given ad libitum access to water and food throughout the in-life phase of the studies. NYX-PCSK9i was formulated in 5% DMSO, 5% Solutol HS15 and 90% saline, and administered IV at 5 mg/kg, PO at 50 mg/kg or SC at 50 mg/kg. At designated timepoints (*t* = 0.083, 0.25, 0.5, 1, 2, 4, 8, and 24 h), the animals were manually restrained, and 110 μl of blood per timepoint was collected into precooled EDTA-K2 tubes via the facial vein. Plasma samples were assayed for their levels of NYX-PCSK9i by LC/MS/MS using an AB SCIEX 6500+ Triple Quad system operating in a positive electrospray mode. Bioavailability was determined by comparing the plasma exposure levels (AUC_last_) of NYX-PCSK9i when given orally with plasma exposure levels obtained via the IV route. Mathematically, this is expressed as *F* (%) = 100% × [(AUC_last, po_ × D_iv_)/(AUC_last, iv_ × D_po_)], where *F* represents the bioavailability, AUC_last, po_ is the area under the curve following NYX-PCSK9i oral administration in h∗ng/ml, AUC_last, iv_ is the area under the curve following NYX-PCSK9i IV administration in h∗ng/ml, D_iv_ is the dose given IV in mg/kg, and D_po_ is the dose in mg/kg given orally.

#### Efficacy studies

Animal experiments were approved by the governmental central committee on animal experiments (AVD5010020172064) and TNO’s animal welfare body (TNO-471 and TNO-482). Female APOE∗3-Leiden.CETP transgenic mice (11–16 weeks of age) were bred at the animal facility of TNO-InnoSer (Leiden, The Netherlands). Female animals were used in this study as they are more responsive to dietary cholesterol and fat than males and therefore typically develop atherosclerosis more rapidly than male APOE∗3-Leiden and APOE∗3-Leiden.CETP mice on cholesterol-containing diets (33, 34). Although measurements of atherosclerosis were not included as part of the present study, females were used with the prospect in mind that atherosclerosis studies will follow. The mice were housed under standard conditions that included 12 h light-dark cycles and access to a WTD and sterilized tap water ad libitum. Body weight, food intake, and clinical signs of behavior were monitored throughout the studies. Mice were fed a semisynthetic WTD [containing 15% saturated fat and 0.15% cholesterol (w/w)]) for a run-in period of 3 weeks, after which mice were matched into groups based on age, body weight, plasma total cholesterol, and triglyceride levels. Time *t* = 0 (weeks) was designated as the time at which mice were matched into groups after the 3-week run-in period in each efficacy study.

In the first efficacy study, mice were divided into three groups of eight to determine whether there was a dose-dependent cholesterol-lowering effect with NYX-PCSK9i treatment. Mice in group 1 received vehicle solution (5% DMSO, 5% Solutol HS15, and 90% saline) twice daily by oral gavage for 28 days. Mice in groups 2 and 3 received NYX-PCSK9i at a dose of 30 and 50 mg/kg respectively, twice daily by oral gavage, for 28 days. Treatment times were around 8:00 am and 4:00 pm each day. Throughout the in-life phase of the study, body weight and food intake (per cage) were measured at regular predetermined intervals while blood samples were collected every 7 days. The last gavage was administered on day 27. On day 28, the mice were sacrificed by CO_2_ asphyxiation, EDTA-plasma was obtained via heart puncture, and the liver was weighed and collected. Throughout the study, plasma cholesterol, triglycerides, HDL-C, lipoprotein profiles, PCSK9 expression levels, aspartate transaminase levels (AST), and alanine transaminase levels (ALT) were measured as described below.

In a subsequent efficacy study, mice were treated identically to the first study with the following modifications. Mice in group 1 received vehicle solution (5% DMSO, 5% Solutol HS15, and 90% saline) twice daily by oral gavage. Mice in group 2 received NYX-PCSK9i at a dose of 50 mg/kg twice daily by oral gavage, mice in group 3 received atorvastatin as a diet admix at a dose of 4.9 mg/kg/day and vehicle solution as per group 1, while mice in group 4 received a combination of both NYX-PCSK9i and atorvastatin using a dose equal to that administered in groups 2 and 3. The study ran for a total of 35 days with blood and tissue collection and analysis performed as described below.

#### Blood collection, plasma lipids, AST, ALT, and lipoprotein analyses and measurement of PCSK9 levels

In both studies, blood samples were collected at weekly intervals via tail vein sampling after fasting the mice for 4 h. Blood samples were centrifugated (4500 *g* at 4°C), and the plasma was collected for the following analyses. Total plasma cholesterol levels were determined using a Cholesterol CHOD-PAP kit according to the manufacturer’s instructions. Plasma HDL-C levels were determined by first precipitating apoB-containing lipids using PEG 6000/glycine and then utilizing the Cholesterol CHOD-PAP kit (35). Non-HDL-C levels were determined by subtracting HDL-C levels from total cholesterol levels (32). Group-pooled lipoprotein profiles were determined by FPLC analysis using an AKTApurifier 10 FPLC system (GE Healthcare, Chicago, IL). Plasma PCSK9 levels were determined using a Mouse PCSK9 Quantikine ELISA kit as per the manufacturer’s protocol. ALT and AST levels of group-pooled samples were measured using the Roche Reflotron Plus System (Mannheim, Germany).

#### Hepatic protein and gene expression analyses

Hepatic LDLR protein levels of mice treated with NYX-PCSK9i and/or atorvastatin were determined by Western blotting as previously reported (29). The transcript levels of select genes in the liver tissue of mice treated with either vehicle or NYX-PCSK9i were analyzed. RNA was isolated from liver tissue using an RNA STAT-60 kit as per the manufacturer’s protocol. RNA concentrations were determined using a NanoDrop 2000 spectrophotometer (Thermo Fisher Scientific, Rockford, IL), and up to 2 μg of RNA was used as a template for cDNA synthesis using a High-capacity RNA-to-cDNA kit according to the manufacturer’s instructions. Quantitative PCR was performed with fluorescein amidite-labeled TaqMan® Assay primers and TaqMan® Fast Universal PCR Master Mix using a QuantStudio 6 Flex Real-Time PCR system (Applied Biosystems). DNA was amplified by applying a standard cycle protocol with an annealing temperature of 60°C and 40 cycles of amplification. Differential transcriptional activity was calculated using the 2^–ΔΔCt^ method.

#### Analyses of hepatic lipid content and fecal bile acid and neutral sterol content

Liver lipid levels were determined in terminal hepatic tissue by high-performance thin-layer chromatography on silica gel plates as previously described (36). Analyses of fecal neutral sterol and bile acid contents were performed by gas chromatographic analysis as previously detailed (37, 38). Per treatment group, two cages of four mice per cage were used, with feces collected twice per cage during the last week of the study. The net cholesterol uptake, or cholesterol balance, was defined as the dietary cholesterol intake (food intake measured weekly) minus the summed fecal output of neutral sterols and bile acids.

#### Statistical analysis

The computer program IBM® SPSS® Statistics 25.0 was used for statistical analyses. The Kolmogorov-Smirnov and Shapiro-Wilk tests were used to test for normal distribution of the data. For nonparametric calculations, a Kruskal-Wallis test for several independent samples was used, followed by a Mann-Whitney U-test for independent samples. For parametric calculations, a one-way ANOVA for multiple comparisons was used, followed by Bonferroni’s posthoc test. A *P*-value ≤ 0.05 was considered statistically significant.

## Results

### NYX-PCSK9i is a newly discovered small-molecule inhibitor of PCSK9 that demonstrates enhanced oral bioavailability

**Fig. 1:**
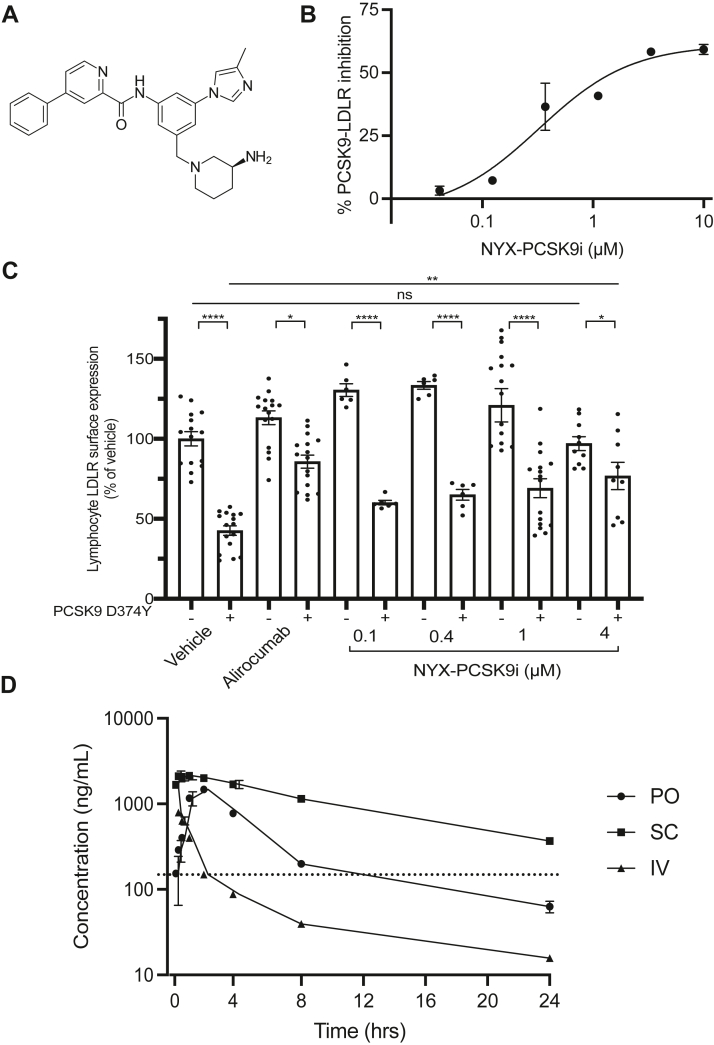
A: The chemical structure of NYX-PCSK9i. B: The impairment of PCSK9 binding with LDLR by NYX-PCSK9i, represented as a function of compound concentration. Data from a single representative experiment is shown with error bars denoting the SEM of duplicate samples. C: NYX-PCSK9i restores the expression of LDLR on the surface of human lymphocytes in the presence of recombinant mutant PCSK9-D374Y. PBMCs from a human donor were incubated with or without recombinant PCSK9-D374Y along with vehicle, 0.1–4 μM NYX-PCSK9i, or alirocumab as indicated. The average level of LDLR expression on the surface of lymphocytes, relative to vehicle-treated controls, is presented. D: The average plasma levels of NYX-PCSK9i in female C57BL/6 mice treated with a single dose of compound via PO (50 mg/kg), SC (50 mg/kg), or IV (5 mg/kg) routes. Plasma concentrations are shown as a function of time (n = 3 animals per administration arm). The *horizontal dotted line* represents the IC_50_ of NYX-PCSK9i as evaluated in the PCSK9-LDLR assay depicted in panel A. The pharmacokinetic properties of NYX-PCSK9i were derived from this data set and are presented in Table 1. In panels C and D: Data represented as mean ± SEM. ns = not significant, ∗*p ≤ 0.05*, ∗∗*p* ˂ 0.01, ∗∗∗∗*p* ˂ 0.0001. PBMC, peripheral blood mononuclear cells; PCSK9, Proprotein convertase subtilisin kexin type 9.

NYX-PCSK9i emerged from a medicinal chemistry campaign to identify analogues of compound 3f (28) with improved potency against the PCSK9-LDLR interaction and enhanced oral bioavailability. NYX-PCSK9i (Fig. 1A) demonstrated submicromolar inhibitory activity against the PCSK9-LDLR interaction in an in vitro biochemical binding assay (IC_50_ = 323 nM, Fig. 1B). Functional inhibition of PCSK9 by NYX-PCSK9i was established in a cellular assay using human lymphocytes that endogenously express LDLR at their surface. Importantly, primary human lymphocytes do not endogenously express or secrete PCSK9 (39). Figure 1C shows that the addition of PCSK9 D374Y to the lymphocytes decreased LDLR surface expression by ∼58% in vehicle-treated control samples, confirming the PCSK9-directed degradation of LDLR. Coincubation of lymphocytes with PCSK9 D374Y and alirocumab, a monoclonal antibody against PCSK9 (12), restored LDLR expression at the cell plasma membrane (Fig. 1C). Lymphocytes that were exposed to PCSK9 D374Y and 4 μM NYX-PCSK9i also demonstrated a large and significant increase in LDLR expression levels relative to controls treated with PCSK9 D374Y and vehicle (Fig. 1C), a result that indicates significant protection of the receptor from PCSK9-directed degradation. Moreover, the protection of LDLR conferred by NYX-PCSK9i at 4 μM was similar in magnitude relative to alirocumab-treated lymphocytes (Fig. 1C), an observation that suggests an equivalence in efficacy between the two PCSK9 inhibitors.

Next, we established the pharmacokinetics of NYX-PCSK9i to determine whether its bioavailability fit the requirements of an orally administered small-molecule inhibitor. Plasma levels of NYX-PCSK9i in wildtype C57BL/6 mice were measured over 24 h following PO, SC (both at 50 mg/kg), or IV (at 5 mg/kg) administration. Figure 1D exhibits the pharmacokinetic profile of NYX-PCSK9i via the three routes, while Table 1 summarizes the key pharmacokinetic properties derived from the data presented in Fig. 1D. It is most noteworthy that NYX-PCSK9i was readily detectable in mouse plasma throughout 24 h following oral delivery of NYX-PCSK9i at 50 mg/kg with an overall exposure of ∼ 8,100 h∗ng/ml (AUC_last_, Table 1). When compared directly with the exposure of NYX-PCSK9i achieved by IV administration (2,006 h∗ng/ml, Table 1), an oral bioavailability of 40% was determined for NYX-PCSK9i, a metric that indicated much-improved bioavailability over compound 3f (28) in C57BL/6 mice. In addition, we noted that the plasma concentrations of NYX-PCSK9i exceeded the PCSK9-LDLR binding IC_50_ value of 323 nM (Fig. 1B) for ∼12 h following oral dosing of the compound (horizontal dotted line, Fig. 1D). Although the pharmacokinetics of NYX-PCSK9i likely differs in WTD-fed APOE∗3-Leiden.CETP transgenic mice used in the efficacy studies, it was rationalized that twice-daily dosing at 50 mg/kg/dose would be a reasonable starting point to initially test the efficacy of NYX-PCSK9i in vivo. We also incorporated a lower dose (30 mg/kg BID) into the regimen for the potential to observe a dose-dependent effect.

**Table 1:** The pharmacokinetic properties of NYX-PCSK9i after dosing female C57BL/6 mice with a single dose via various routes

Route	CL (L/h/kg)	Vss (L/kg)	T_1/2_ (h)	AUC last (h∗ng/ml)	AUC Infinity (h∗ng/ml)	MRT Infinity (h)
50 mg/kg PO			6.29	8131	8702	7.07
50 mg/kg SC			9.11	25,518	30,366	12.2
5 mg/kg IV	2.29	14.7	7.48	2017	2186	6.42

These data were derived from the data set presented in Fig. 1D.

Pharmacokinetic parameters are defined as follows: CL, clearance of compound; V_ss_, volume of distribution at equilibrium; T_1/2_, terminal half-life; AUC_last_, area under the blood concentration-time curve from t = 0 to the last measurable time point; AUC_∞_, area under the blood concentration-time curve from t = 0 to infinity, MRT_∞_, mean residence time extrapolated to infinity.

### NYX-PCSK9i demonstrates significant cholesterol-lowering properties in APOE∗3-Leiden.CETP hyperlipidemic mice

**Fig. 2:**
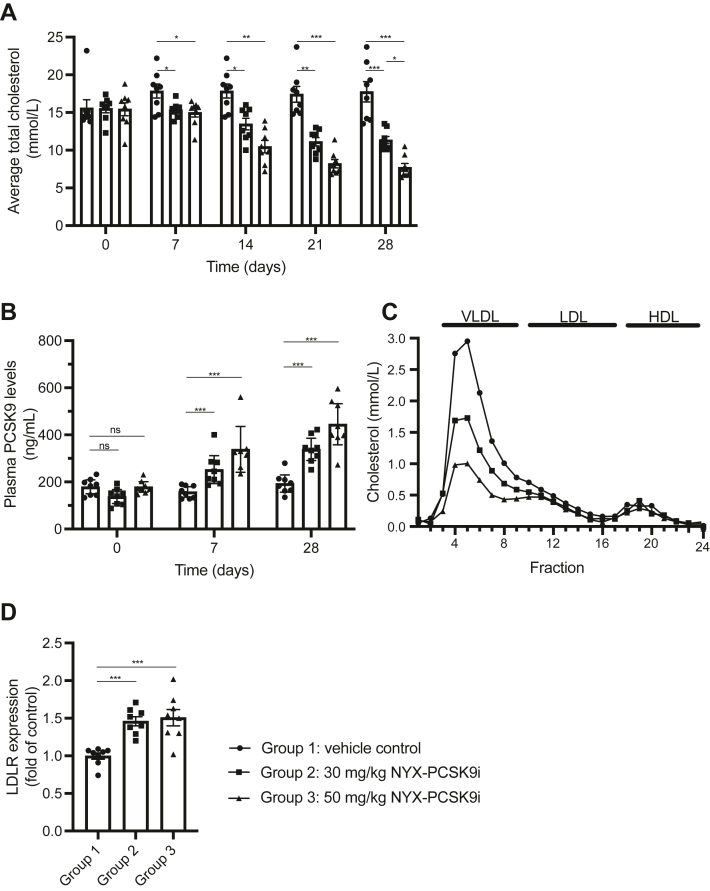
NYX-PCSK9i significantly reduces plasma total cholesterol and elevates plasma total PCSK9 levels in a dose-dependent fashion. Female APOE∗3-Leiden.CETP mice were dosed orally with vehicle, 30 mg/kg, or 50 mg/kg NYX-PCSK9i BID for 28 days. (A) Plasma total cholesterol and (B) plasma total PCSK9 levels were measured at weekly intervals throughout the study, with a dose-dependent change in both parameters. Error bars represent the SEM derived from eight plasma samples each taken from an individual mouse. C: The lipoprotein profile of each treatment cohort was evaluated by FPLC following 28 days of treatment. The cholesterol levels of each fraction were determined and are shown as a function of fraction number. A total of eight samples from each cohort were pooled for FPLC analysis. D: Liver tissue was collected on day 28 and analyzed for LDLR protein expression by Western blotting. LDLR expression levels were controlled for sample loading and normalized to vehicle-only controls. PCSK9, Proprotein convertase subtilisin kexin type 9; FPLC, fast protein liquid chromatography; HDL, high density lipoprotein; LDL, low density lipoprotein; VLDL, very low density lipoprotein. ns = not significant, ∗*p* ˂ 0.05, ∗∗*p* ˂ 0.01, ∗∗∗*p* ˂ 0.001. In panels A, B, D: Data represented as mean ± SEM (n = 8 animals per group).

Throughout the first efficacy study, vehicle-treated APOE∗3-Leiden.CETP mice receiving a WTD exhibited plasma total cholesterol levels ranging from 15 to 18 mM (or 580–696 mg/dl) (Fig. 2A). Treatment with 30 and 50 mg/kg NYX-PCSK9i significantly decreased plasma total cholesterol relative to vehicle-treated controls from day 7 of treatment onward in a time-dependent and dose-dependent manner (Fig. 2A). The maximal reduction in total cholesterol occurred on day 28 of treatment and was - 36% and - 57% for 30 and 50 mg/kg dose arms, respectively, relative to vehicle-treated controls (Fig. 2A). Notably, the 57% decrease in total cholesterol induced by 50 mg/kg NYX-PCSK9i on day 28 represents a reduction of 10.1 mM (390 mg/dl) cholesterol in absolute terms (Fig. 2A). The vast bulk of the cholesterol lost with NYX-PCSK9i treatment was non-HDL-C (supplemental Table S1), and most prominently in fractions containing VLDL/VLDL remnants and LDLs as displayed by the lipoprotein profile in Fig. 2C. In contrast, there was no consistent reduction in HDL-C levels by treatment with NYX-PCSK9i (supplemental Table S2). Mean plasma triglyceride levels were essentially unchanged with NYX-PCSK9i treatment (supplemental Table S3).

In parallel with cholesterol measurements, we assayed mouse plasma for total PCSK9 levels. Treatment with 30 and 50 mg/kg NYX-PCSK9i significantly and dose-dependently elevated plasma PCSK9 protein levels relative to vehicle-treated controls (Fig. 2B). Plasma PCSK9 protein levels were elevated from the first measurement on day 7 and reached a maximal 2-fold increase on day 28 with 50 mg/kg NYX-PCSK9i (Fig. 2B). Despite an increase in plasma total PCSK9 levels with NYX-PCSK9i exposure, hepatic LDLR protein levels were significantly increased with 30 mg/kg and 50 mg/kg NYX-PCSK9i treatments (46% and 51%, respectively) on day 28 of the study, although no dose-dependent response was evident (Fig. 2D).

Markers of general health, including body weight and food intake, were not significantly altered in any treatment groups relative to vehicle-treated controls (supplemental Tables S4 and S5), indicating good tolerability of NYX-PCSK9i. Similarly, plasma levels of the liver enzymes AST (supplemental Table S6) and ALT (supplemental Table S7) in NYX-PCSK9i-treated mice remained stable, compared to vehicle-treated controls.

### NYX-PSCK9i combines effectively with atorvastatin to further reduce plasma cholesterol

**Fig. 3:**
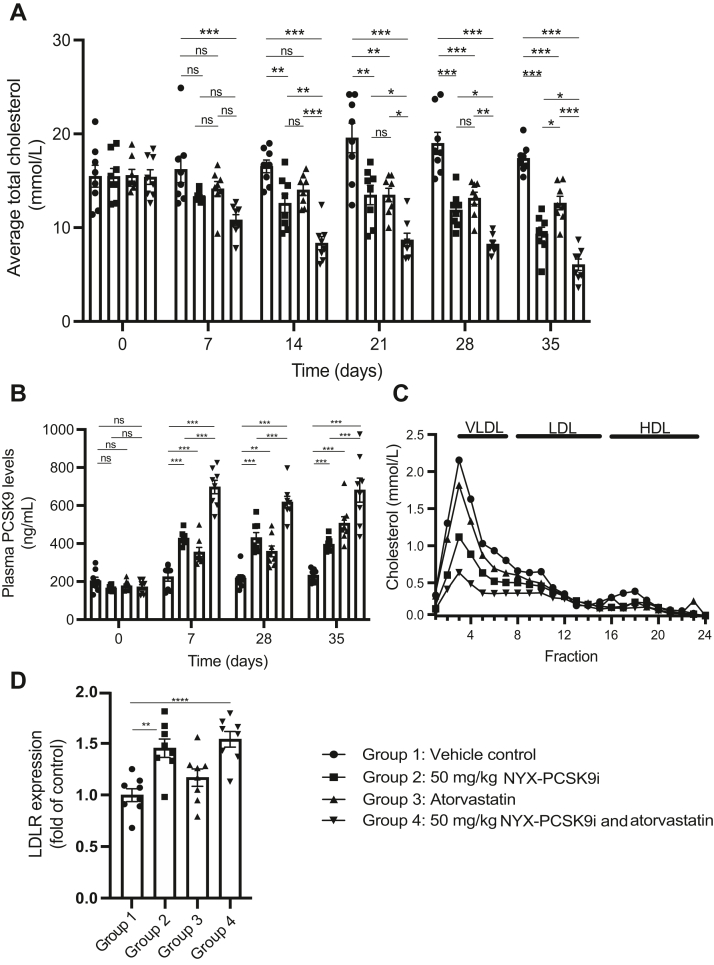
NYX-PCSK9i enhances the efficacy of atorvastatin in lowering plasma total cholesterol levels. Female APOE∗3-Leiden.CETP mice were treated orally with vehicle, 50 mg/kg NYX-PCSK9i BID, 4.9 mg/kg/day atorvastatin, or a combination of both for 35 days. Plasma was collected weekly and assayed for (A) total cholesterol and (B) total PCSK9 levels. C: At the end of the 35-days treatment period, plasma samples were group-pooled, and their lipoprotein profiles analyzed by FLPC. The cholesterol concentrations of each fraction were determined and are represented as a function of the fraction number. D: Liver tissue was collected on day 35 and analyzed for LDLR protein expression by Western blotting. LDLR expression levels were controlled for sample loading and normalized to vehicle-only controls. FPLC, fast protein liquid chromatography; HDL, high density lipoprotein; LDL, low density lipoprotein; PCSK9, Proprotein convertase subtilisin kexin type 9; VLDL, very low density lipoprotein; ns = not significant, ∗*p* ˂ 0.05, ∗∗*p* ˂ 0.01, ∗∗∗*p* ˂ 0.001, ∗∗∗∗*p* ˂ 0.0001. In panels A, B, D: Data represented as mean ± SEM (n = 8 animals per group).

Statins are highly effective in reducing primary and secondary cardiovascular events; however, they are known to increase serum PCSK9 levels (40). With this in mind, we sought to test whether NYX-PCSK9i may enhance the activity of atorvastatin in lowering plasma cholesterol levels. Treatment of APOE∗3-Leiden.CETP mice for 5 weeks with vehicle again demonstrated plasma total cholesterol levels of approximately 16–20 mM throughout the study (Fig. 3A). Compared to the vehicle control–treated mice, monotherapy with 50 mg/kg NYX-PCSK9i significantly decreased plasma total cholesterol from day 14 onwards (Fig. 3A). While the maximum reduction in plasma total cholesterol by NYX-PCSK9i was achieved on day 35 (−46%, Fig. 3A), we observed a further decrease in combination with 4.9 mg/kg atorvastatin treatment (−65%, Fig. 3A). Atorvastatin monotherapy reduced plasma total cholesterol by 27% at the same timepoint (Fig. 3A), indicating that the combination was roughly additive in suppressing plasma total cholesterol levels on day 35 (Fig. 3A). Indeed, the additive nature of the combination in reducing total cholesterol was evident on day 7 and was sustained throughout the study’s entirety until day 35 (Fig. 3A).

Consistent with the lipoprotein profile associated with NYX-PCSK9i monotherapy of the first efficacy study (Fig. 2C), NYX-PCSK9i treatment alone or in combination with atorvastatin most prominently suppressed levels of cholesterol associated with non-HDL lipoproteins (Fig. 3C). The reduction in non-HDL-C was also most pronounced in mice treated with the combination (Fig. 3C and supplemental Table S8). Both mono and combination therapies had little effect on HDL-C or mean plasma triglyceride levels (Fig. 3C, supplemental Tables S9 and S10).

Much like the first efficacy study (Fig. 2B), NYX-PCSK9i monotherapy significantly elevated total PCSK9 levels by approximately 2-fold from day 7, a response that was sustained for the remaining 4 weeks (Fig. 3B). Atorvastatin treatment alone induced a similar increase in PCSK9 plasma levels; however, the increase in magnitude was generally lower (Fig. 3B). The combination therapy increased total plasma PCSK9 levels more than either monotherapy alone, with the total elevation in PCSK9 levels roughly equivalent to the sum of each monotherapy (Fig. 3B). Atorvastatin had no significant effect on hepatic LDLR protein expression; however, both NYX-PCSK9i monotherapy and the combination resulted in a significant increase in hepatic LDLR protein expression, by 45% and 54%, respectively (Fig. 3D). Again, no adverse effects in any of the treatment groups were observed in measured aspects of general animal health and liver function (supplemental Tables S11–S14).

### NYX-PCSK9i modulates the hepatic transcriptional activity of select genes

**Fig. 4:**
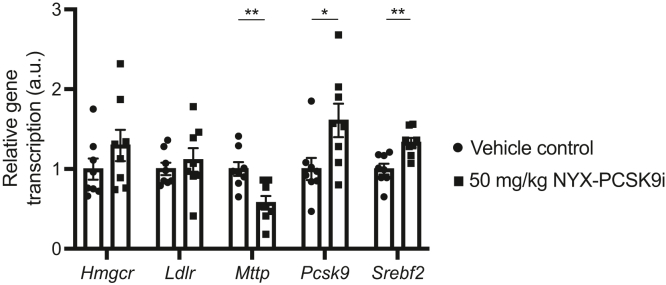
NYX-PCSK9i modulates the hepatic transcriptional activity of specific genes involved in cholesterol metabolism. Female APOE∗3-Leiden.CETP mice were treated orally with vehicle or 50 mg/kg NYX-PCSK9i BID each day for 5 weeks. On day 35, liver tissue was assessed for changes in the transcription of select genes using qPCR. The relative expression of each gene is shown in arbitrary units (a.u) and normalized to vehicle-only controls. ∗*p* ˂ 0.05, ∗∗*p* ˂ 0.01. Data represented as mean ± SEM (n = 8 animals per group). qPCR, quantitative polymerase chain reaction.

To further probe how NYX-PCSK9i affected in vivo changes in hepatic LDLR and plasma PCSK9 expression, transcriptional analysis of *Pcsk9*, *Ldlr*, and several other genes involved in cholesterol metabolism was performed. Transcription of *Hmgcr* in the liver tissue of APOE∗3-Leiden.CETP mice treated with 50 mg/kg NYX-PCSK9i for 5 weeks was unchanged relative to vehicle-control–treated animals (Fig. 4). Expression levels of the *Srebf2* transcript were significantly upregulated by 33% with NYX-PCSK9i treatment (Fig. 4), an observation that is consistent with an increase in the transcription of *Pcsk9* by 61% (Fig. 4). On day 35 of treatment, hepatic transcription levels of *Ldlr* were unaffected by NYX-PCSK9i treatment relative to vehicle-treated mice (Fig. 4). Finally, NYX-PCSK9i significantly suppressed the expression of *Mttp* transcripts by 43% relative to vehicle-treated controls (Fig. 4).

### NYX-PCSK9i depletes hepatic cholesteryl ester levels and promotes fecal cholesterol elimination

**Fig. 5:**
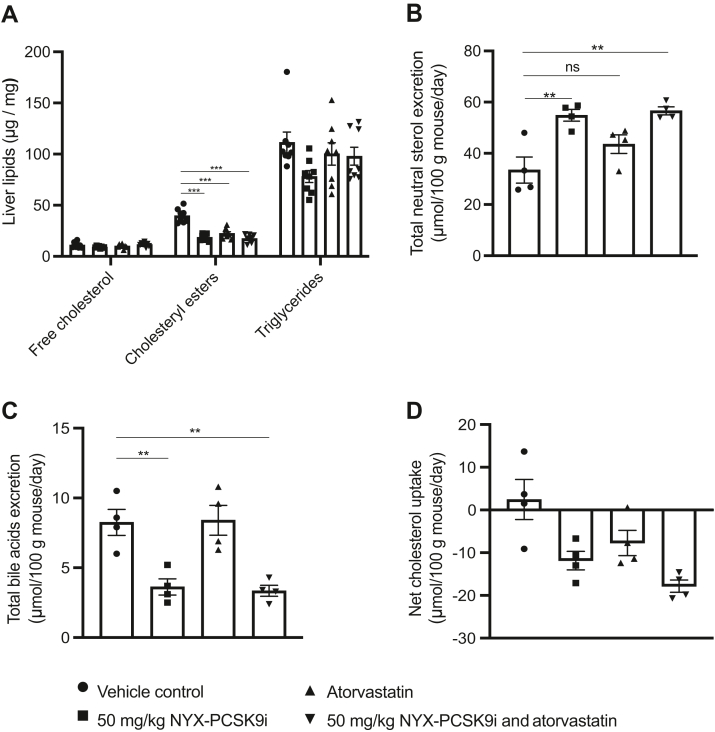
NYX-PCSK9i, atorvastatin, and their combination promote cholesterol excretion from mice. Female APOE∗3-Leiden.CETP mice were treated orally with vehicle, 50 mg/kg NYX-PCSK9i BID, 4.9 mg/kg/day atorvastatin, or a combination of both for 35 days A: Liver tissue was collected on day 35 and analyzed for levels of free cholesterol, cholesteryl esters, and triglycerides. B-D: Feces were collected during the final week of the study. Two samples were collected per cage of 4 mice with 48–72 h between collection points, therefore each data point represents one collection point per cage. Feces were analyzed for either (B) total neutral sterols or (C) total bile acids. D: The cholesterol balance as shown was calculated as the difference between dietary cholesterol intake and the combined excretion of bile acids and neutral sterols via the feces. ns = not significant, ∗∗*p* ˂ 0.01, ∗∗∗*p* ˂ 0.001 (Data represented as mean ± SEM, A: n = 8 animals per group; B–D: n = 2 cages of 4 mice per group).

Given the increase in hepatic LDLR protein levels with NYX-PCSK9i treatment (Figs. 2D and 3D), we took the opportunity to determine any changes in hepatic cholesterol levels. Measurement of liver lipid levels showed that in all three treatment groups, free cholesterol and triglycerides were unchanged by the end of the study, whilst a significant reduction in cholesteryl esters was observed relative to vehicle-treated controls (Fig. 5A). We saw no significant difference between the monotherapies and the combination treatment (Fig. 5A). As early exploratory work to further investigate the effect of NYX-PCSK9i treatment on cholesterol elimination, we examined fecal excretion of bile acids and neutral sterols. NYX-PCSK9i treatment, as a monotherapy or in combination with atorvastatin, resulted in significantly increased fecal neutral sterols (64% and 69%, respectively Fig. 5B), of which more than 95% was cholesterol for both NYX-PCSK9i monotherapy and combination treatments (data not shown). In contrast, NYX-PCSK9i treatment alone or in combination with atorvastatin significantly reduced bile acid excretion in feces by 57% and 60%, respectively (Fig. 5C). The cholesterol balance, calculated as the difference between dietary cholesterol intake and the combined excretion of bile acids and neutral sterol via the feces, was negative in animals treated with NYX-PCSK9i or atorvastatin monotherapies (Fig. 5D). Animals treated with the combination exhibited the greatest net cholesterol excretion with the net lowering in cholesterol approximately equivalent to the sum of the reductions associated with each monotherapy (Fig. 5D).

## Discussion

Following its discovery as part of a medicinal chemistry campaign to identify an orally bioavailable small-molecule inhibitor of PCSK9, NYX-PCSK9i (Fig. 1A) demonstrated potent disruption of the PCSK9-LDLR interaction in vitro (IC_50_ = 323 nM, Fig. 1B). NYX-PCSK9i is a structural analog of an earlier proof-of-concept molecule designated compound 3f and presumably binds to the catalytic domain of PCSK9 via the same binding groove detailed by Evison *et al.* (28), although this is yet to be confirmed experimentally. While it is clear that NYX-PCSK9i disrupts the interaction of PCSK9 with LDLR (Fig. 1B), it is currently unknown whether the compound inhibits the interaction of PCSK9 with other protein binding partners such as CD36 (41), and this is an area worthy of further investigation. Given its inhibitory activity against the PCSK9-LDLR interaction and its improved oral bioavailability (Fig. 1D) over compound 3f (28), NYX-PCSK9i was tested for its lipid-lowering properties as a monotherapy and in combination with atorvastatin in the APOE∗3-Leiden.CETP murine model of hyperlipidemia. To the best of our knowledge, this is the first study investigating the effect of combining an orally bioavailable small-molecule PCSK9 inhibitor with statin treatment in vivo.

We chose the APOE∗3-Leiden.CETP mouse model of hyperlipidemia to test NYX-PCSK9i efficacy as it is representative of human lipoprotein metabolism with respect to plasma lipoprotein levels, lipoprotein profiles, and has also been shown to be responsive to lipid-modulating drugs currently used in the clinic (42). The model is particularly sensitive to lipid-modulating drugs in the context of disease settings, such as atherosclerosis and metabolic syndrome (32, 43, 44). As in humans, APOE∗3-Leiden.CETP transgenic mice respond to treatment with these FDA-approved drugs with reductions in apoB-containing lipoproteins, and some of the mechanisms are similar to those observed in humans. This supports the translational use of this mouse model. We chose to apply an atorvastatin dose that reduced total cholesterol by 20%–30% in the present study (Fig. 3A), a decrease that is in accordance with previous clinical trials of atorvastatin in humans and commonly observed in the general population (45). Therefore, the significant dose-dependent decrease in plasma total cholesterol (and non-HDL-C) observed with NYX-PCSK9i treatment (Figs. 2A, C and 3A, C) may be predictive of translational efficacy in humans. Indeed, we are currently exploring this hypothesis further by progressing with this class of compounds toward first-in-human trials.

The significant reduction in plasma total cholesterol by NYX-PCSK9i monotherapy across two separate studies (46%–57%, Figs. 2A and 3A) is a particular highlight of this report. The bulk of the total cholesterol lost was non-HDL-C (Figs. 2C, 3C, supplemental Tables S1 and S7), a particularly encouraging result given the link between elevated plasma non-HDL-C and CVD (1, 2, 46, 47). To provide some context around this result, alirocumab demonstrated a decrease in plasma total cholesterol of 37%–46% in APOE∗3-Leiden.CETP mice that received the antibody via weekly injections for 18 weeks (29). In addition, the lipoprotein profile of alirocumab-treated APOE∗3-Leiden.CETP mice showed that most of the cholesterol lowering was associated with non-HDL (29), much like NYX-PCSK9i (Figs. 2C and 3C). Although a longer-term study is yet to be initiated, the similarities in efficacy shared by alirocumab and NYX-PCSK9i in the same mouse model of hyperlipidemia add weight to NYX-PCSK9i′s viability as an alternative to monoclonal PCSK9 antibodies in the clinic.

There is strong evidence to support on-target action by NYX-PCSK9i. Firstly, NYX-PCSK9i functioned to protect LDLR protein on the surface of human lymphocytes from PCSK9-directed degradation (Fig. 1C), a result consistent with the action of alirocumab and evolocumab in this ex vivo assay system (39). Secondly, and perhaps most importantly, hepatic levels of LDLR protein were significantly elevated in APOE∗3-Leiden.CETP mice treated with NYX-PCSK9i (Figs. 2D and 3D). We have shown that NYX-PCSK9i did not simply upregulate hepatic LDLR expression by increasing transcription of the gene in liver tissue (Fig. 4). Rather, the elevated hepatic LDLR levels associated with NYX-PCSK9i exposure likely reflect a posttranscriptional mechanism of action, namely that of protection of LDLR from PCSK9-directed degradation. It is noteworthy that a similar increase in hepatic LDLR expression occurred following alirocumab treatment of APOE∗3-Leiden.CETP mice (29). Finally, a robust increase in circulating plasma levels of total PCSK9 (Figs. 2B and 3B) with NYX-PCSK9i exposure also implicates on-target action by NYX-PCSK9i. Similar increases in plasma PCSK9 have been described in multiple studies that include the application of evolocumab in APOE∗3-Leiden.CETP mice (30) and an experimental anti-PCSK9 antibody designated 1B20 in CETP/LDLR-hemi mice and rhesus monkeys (48). Elevated plasma PCSK9 levels (Figs. 2B and 3B) may reflect impaired hepatic uptake of PCSK9 as a consequence of LDLR-PCSK9 inhibition by NYX-PCSK9i. Although the NYX-PCSK9i–induced reduction in non-HDL-C coincided with increased hepatic LDLR protein expression, elucidation of the mechanism underlying the VLDL lowering will require studies of VLDL production/secretion and of LDL clearance.

It is noteworthy that plasma total cholesterol levels exhibited a dose-dependent decrease with NYX-PCSK9i monotherapy at day 28 (Fig. 2A), yet hepatic LDLR protein levels failed to show a similar dose response at the same time point (Fig. 2D). The inconsistency between these two parameters is suggestive that there may be an additional, distinct mechanism of action by NYX-PCSK9i at play that does not involve increasing hepatic LDLR protein levels beyond those observed in Fig. 2D. Potentially, this mechanism may involve the inhibition of PCSK9 at a site of action distinct from the liver (e.g., the intestines, discussed further below) and/or a mechanism that is presently unknown and independent of PCSK9 function. Work is ongoing to resolve this potential additional mechanism of action.

The significant decrease in hepatic transcription of *Mttp* observed in NYX-PCSK9i–treated mice (Fig. 4) warrants singular mention. Microsomal triglyceride transfer protein functions as a chaperone by facilitating the assembly of lipids into apoB-containing lipoproteins that include VLDLs (49). The decrease in hepatic *Mttp* transcription may reflect a decrease in VLDL production by the liver, a notion that is consistent with the suppression of plasma non-HDL-C by NYX-PCSK9i (Figs. 2C and 3C). The potential involvement of microsomal triglyceride transfer protein in lipid-lowering by NYX-PCSK9i is the subject of ongoing investigations.

For many individuals at risk of CVD, statin monotherapy is often insufficient to achieve their LDL-C targets (50, 51). At a molecular level, statin exposure activates the expression of both LDLR and PCSK9 in tandem, predominantly through the activation of their shared regulator SREBP-2 (52). An outcome of this co-activation is that atorvastatin treatment directly causes an increase in serum PCSK9 in humans (53), restricting the effectiveness of the statin. We surmised that a combination therapy consisting of atorvastatin and NYX-PCSK9i may circumvent the inhibitory effect of PCSK9 on LDLR expression levels, thereby further reducing LDL-C plasma levels. While the addition of NYX-PCSK9i to atorvastatin did not further elevate hepatic LDLR protein levels relative to NYX-PCSK9i monotherapy controls (Fig. 3D), NYX-PCSK9i significantly enhanced the lipid-lowering activity of atorvastatin, with the combination additively suppressing total cholesterol throughout the 35-days study (Fig. 3A). The absence of a clear additive response in hepatic LDLR protein levels despite a robust additive decrease in plasma total cholesterol levels by the combination is worth highlighting as it indicates that the combination may be working to lower cholesterol via an additional mechanism distinct from hepatic LDLR-mediated clearance of plasma non-HDL-C. It has been demonstrated that both atorvastatin (54) and rosuvastatin (55) predominantly function to decrease plasma total cholesterol levels via a reduction in VLDL production and secretion in APOE∗3-Leiden mice while the involvement of the LDLR is less important than in humans. The additivity observed in lipid lowering by the combination of NYX-PCSK9i and atorvastatin in APOE∗3-Leiden mice (Fig. 3A) may therefore represent the sum of these two independent mechanisms; however, further work will be required to fully address this question.

The additive nature of this interaction in suppressing plasma total cholesterol and non-HDL-C levels (Fig. 3A, C, respectively) holds great promise for the inhibition of atherosclerosis in the longer term. Kühnast *et al.* demonstrated that the addition of alirocumab to atorvastatin treatment in APOE∗3-Leiden.CETP mice enhanced the beneficial effects of atorvastatin in terms of reducing atherosclerotic lesion size and severity over 18 weeks (29). Since atherosclerotic lesion size positively correlates with plasma total cholesterol levels in APOE∗3-Leiden.CETP mice (29), it would be of value to determine in future studies if the combination of atorvastatin and NYX-PCSK9i would similarly reduce lesion size over an extended treatment period of 18 weeks.

Given the decrease in plasma non-HDL-C observed in APOE∗3-Leiden.CETP mice treated with NYX-PCSK9i (Figs. 2C and 3C and supplemental Tables S1 and S8), it was reasonable to anticipate changes in hepatic cholesterol composition. Surprisingly, NYX-PCSK9i treatment was not associated with an increase in hepatic lipid content, rather hepatic cholesteryl ester levels were depleted while levels of free cholesterol were largely unchanged (Fig. 5A). The majority of free cholesterol is contained within cellular membranes (56) while the pool of regulatory cellular free cholesterol is tightly regulated since free cholesterol is toxic to cells and therefore immediately esterified (57). The amount of cholesteryl ester is therefore a more sensitive marker of cholesterol depletion or overload than free cholesterol (58). Interestingly, there was no additive response with respect to hepatic cholesteryl ester levels by the combination of atorvastatin and NYX-PCSK9i at day 35 of treatment (Fig. 5A) despite a clear additive response in the lowering of total plasma cholesterol levels by the combination at the same timepoint (Fig. 3A). The reason for this is unclear at present; however, it is conceivable that a limit of depletion of cholesteryl ester in the liver has been reached. Additionally, it is also possible that with longer treatment, an additive response may yet be observed. Investigations into this will be incorporated into a longitudinal atherosclerotic follow-up study.

The depletion of hepatic cholesterol levels by NYX-PCSK9i monotherapy is also very likely reflected by an increase in the hepatic transcript levels of *Pcsk9* and *Srebf2* (Fig. 4). In cells depleted of cholesterol, it is well established that the protein product of *Srebf2*, SREBP-2, activates the expression of a range of genes involved in cholesterol biosynthesis and transport (59, 60). Both *Pcsk9* and *Ldlr* are target genes of SREBP-2 transactivation (60, 61); however, it was noted that only *Pcsk9* transcription was significantly upregulated by NYX-PCSK9i monotherapy while levels of the *Ldlr* transcript were unchanged (Fig. 4). The rationale for this apparent discrepancy presently unknown.

In addition to the depletion of hepatic cholesteryl ester content with NYX-PCSK9i monotherapy, fecal bile acid content was decreased (Fig. 5C) and fecal neutral sterol levels were enhanced (Fig. 5B) by NYX-PCSK9i exposure. Changes of this nature are surprising for a PCSK9 inhibitor as the anti-PCSK9 monoclonal antibody alirocumab affected none of these parameters in the same APOE∗3-Leiden.CETP mouse model (29). Consistent with this, an independent study reported that hepatic cholesteryl ester and fecal cholesterol levels were no different between wild-type and loss-of-function PCSK9-Y119X mice (62), an observation that suggests no function for PCSK9 in mediating these physiological processes. In contrast, multiple other studies have identified and detailed a role for PCSK9 in cholesterol excretion (63, 64). For example, Le May *et al.* demonstrated that PCSK9 repressed trans-intestinal cholesterol excretion using PCSK9 knockout mice (64). It is conceivable that NYX-PCSK9i may relieve the repression of PCSK9 on trans-intestinal cholesterol excretion, thereby promoting the excretion of fecal cholesterol (Fig. 5B) and facilitating a net reduction in cholesterol intake (Fig. 5D). Further work is required to fully elucidate the mechanisms of action responsible for the net reduction in cholesterol and changes in bile acid excretion observed in the feces with NYX-PCSK9i treatment (Fig. 5B–D).

The present study demonstrates that an oral small-molecule PCSK9 inhibitor, NYX-PCSK9i, dose-dependently decreases plasma total cholesterol in hyperlipidemic APOE∗3-Leiden.CETP mice. The combination of NYX-PCSK9i with atorvastatin further suppresses total cholesterol level, highlighting the possibility to explore combination therapy in humans. Importantly, the cholesterol carried by non-HDLs was most susceptible to suppression by NYX-PCSK9i, and all measured in vivo markers of health remained unchanged compared to vehicle control–treated mice. Thus, NYX-PCSK9i has emerged as a potential new therapy for hypercholesterolemia with NYX-PCSK9i and fellow compounds of this class now progressing towards the clinic to further explore their efficacy.

## Data Availability

All data described in the manuscript are either located within the manuscript and supplementary data or can be requested from the corresponding author Benny J. Evison via +61,498,336,021, benny.evison@nyrada.com

## Supplemental Data

This article contains supplemental data.

## Conflict of Interest

A. K. S., J. P., J. B., and B. J. E. are employees of Nyrada Inc. J. T. P. and G. L. are members of the Scientific Advisory Board of Nyrada Inc. J. T. P., G. L., J. B., and B. J. E. have share/stock options in Nyrada Inc. A. K. S., P. A. C., J. T. P., J. B., and B. J. E. are listed as inventors on the patent that discloses NYX-PCSK9i and related compounds. G. S., P. A. C., E. J. P., N. K., G. L., K. C., A. K. J., and H. M. G. P. are employees or members of their respective companies or laboratories who were contracted and/or consulted by Nyrada Inc. to perform specific research activities.
