# Supplementary material for: A Novel, Orally Bioavailable, Small-Molecule Inhibitor of PCSK9 With Significant Cholesterol-Lowering Properties In Vivo
Source: J Lipid Res. 2022 Oct 6;63(11):100293. doi: 10.1016/j.jlr.2022.100293 (PMC9646673; doi:10.1016/j.jlr.2022.100293)
Supplement: Supplemental data [file mmc1.docx]

**Supplemental data**

**Methods**

Representative chemical characterisation data (1H-NMR and LCMS spectra) for NYX-PCSK9i is provided below.

**Results**

**Table S1**: Mean plasma non-HDL cholesterol levels were largely unchanged with NYX-PCSK9i treatment. Numbers in **bold** are significantly different, compared to control.

|  | Plasma non-HDL- cholesterol (mmol/L; mean ± SEM) | | |
| --- | --- | --- | --- |
| Time (days) | 0 | 14 | 28 |
| Group 1: Vehicle control | 14.8 ± 3.1 | 16.6 ± 3.0 | 16.6 ± 3.7 |
| Group 2: 30 mg/kg NYX-PCSK9i | 14.7 ± 1.6 | **13.1 ± 2.2** | **10.5 ± 1.4** |
| Group 3: 50 mg/kg NYX-PCSK9i | 14.6 ± 2.5 | **9.8 ± 2.4** | **6.8 ± 1.5** |

**Table S2:** Mean plasma HDL-cholesterol levels were largely unchanged with NYX-PCSK9i treatment. Numbers in **bold** are significantly different, compared to control.

|  | Plasma HDL- cholesterol (mmol/L; mean ± SEM) | | |
| --- | --- | --- | --- |
| Time (days) | 0 | 14 | 28 |
| Group 1: Vehicle control | 0.8 ± 0.2 | 0.8 ± 0.1 | 1.1 ± 0.3 |
| Group 2: 30 mg/kg NYX-PCSK9i | 0.9 ± 0.2 | **0.4 ± 0.1** | **0.8 ± 0.2** |
| Group 3: 50 mg/kg NYX-PCSK9i | 0.8 ± 0.1 | 0.6 ± 0.2 | 0.9 ± 0.2 |

**Table S3:** Mean plasma triglyceride levels were largely unchanged with NYX-PCSK9i treatment. Numbers in **bold** are significantly different, compared to control.

|  | Plasma triglycerides (mmol/L; mean ± SD) | | | | |
| --- | --- | --- | --- | --- | --- |
| Time (days) | 0 | 7 | 14 | 21 | 28 |
| Group 1: Vehicle control | 6.1 ± 1.8 | 4.8 ± 2.2 | 4.6 ± 1.5 | 4.0 ± 1.3 | 5.2 ± 1.9 |
| Group 2: 30 mg/kg NYX-PCSK9i | 5.7 ± 2.2 | 7.0 ± 2.0 | **8.9 ± 1.8** | **6.1 ± 1.5** | 5.7 ± 1.9 |
| Group 3: 50 mg/kg NYX-PCSK9i | 6.1 ± 1.9 | 5.9 ± 2.0 | 5.2 ± 1.7 | 4.2 ± 1.3 | 4.1 ± 1.2 |

**Table S4:** No significant change in mean body weight of mice treated with varying concentrations of NYX-PCSK9i was observed across 4 weeks of treatment

|  | Body weight (mean ± SD) | | | | |
| --- | --- | --- | --- | --- | --- |
| Time (days) | 0 | 7 | 14 | 21 | 28 |
| Group 1: Vehicle control | 22.3 ± 2.5 | 21.5 ± 2.2 | 21.5 ± 2.2 | 21.5 ± 2.1 | 22.0 ± 2.5 |
| Group 2: 30 mg/kg NYX-PCSK9i | 21.9 ± 1.3 | 22.0 ± 1.6 | 22.3 ± 1.7 | 22.2 ± 1.2 | 22.0 ± 1.1 |
| Group 3: 50 mg/kg NYX-PCSK9i | 22.2 ± 1.6 | 21.8 ± 1.3 | 21.8 ± 1.1 | 21.5 ± 1.4 | 21.6 ± 1.1 |

**Table S5:** No significant change in mean food intake of mice treated with varying concentrations of NYX-PCSK9i was observed across 4 weeks of treatment

|  | Food intake (mean ± SD) | | | | |
| --- | --- | --- | --- | --- | --- |
| Time (days) | 0 | 7 | 14 | 21 | 28 |
| Group 1: Vehicle control | 3.3 ± 0.3 | 2.4 ± 0.3 | 2.6 ± 0.3 | 2.5 ± 0.2 | 2.6 ± 0.2 |
| Group 2: 30 mg/kg NYX-PCSK9i | 3.3 ± 0.3 | 2.7 ± 0.3 | 2.6 ± 0.6 | 2.4 ± 0.2 | 3.0 ± 0.1 |
| Group 3: 50 mg/kg NYX-PCSK9i | 3.3 ± 0.3 | 2.6 ± 0.0 | 2.9 ± 0.0 | 2.7 ± 0.0 | 2.7 ± 0.1 |

**Table S6:** No change in group-pooled levels of the liver enzyme AST in mice treated with varying concentrations of NYX-PCSK9i was detected across 4 weeks of treatment

|  | Plasma AST (U/L) | | | | |
| --- | --- | --- | --- | --- | --- |
| Time (days) | 0 | 7 | 14 | 21 | 28 |
| Group 1: Vehicle control | 159.6 | 142.4 | 124.4 | 112.4 | 96 |
| Group 2: 30 mg/kg NYX-PCSK9i | 185.2 | 175.2 | 158.8 | 156.8 | 84 |
| Group 3: 50 mg/kg NYX-PCSK9i | 170.8 | 181.2 | 147.6 | 120.4 | 107 |

**Table S7:** No change in group-pooled levels of the liver enzyme ALT in mice treated with varying concentrations of NYX-PCSK9i was detected across 4 weeks of treatment

|  | Plasma ALT (U/L) | | | | |
| --- | --- | --- | --- | --- | --- |
| Time (days) | 0 | 7 | 14 | 21 | 28 |
| Group 1: Vehicle control | 70.4 | 64.8 | 59.6 | 48 | 40 |
| Group 2: 30 mg/kg NYX-PCSK9i | 76.8 | 69.6 | 70.0 | 55.6 | 35.6 |
| Group 3: 50 mg/kg NYX-PCSK9i | 70.0 | 74.8 | 56 | 47.6 | 38 |

**Table S8:** Mean plasma non HDL-cholesterol levels after treatment with NYX-PCSK9i, atorvastatin, or the combination of both. Numbers in **bold** are significantly different, compared to control.

|  | Plasma non- HDL-cholesterol (mmol/L) | | | |
| --- | --- | --- | --- | --- |
| Time (days) | 0 | 14 | 28 | 35 |
| Group 1: Vehicle control | 14.5 ± 3.3 | 15.1 ± 3.9 | 15.3 ± 1.9 | 18.7 ± 4.4 |
| Group 2: 50 mg/kg NYX-PCSK9i | 14.6 ± 2.5 | 12.7 ± 0.6 | **11.8 ± 2.8** | **12.8 ± 2.8** |
| Group 3: Atorvastatin control | 14.7 ± 1.8 | 13.1 ± 2.2 | 12.8 ± 1.6 | 15.3 ± 1.9 |
| Group 4: 50 mg/kg NYX-PCSK9i and atorvastatin | 14.5 ± 2.2 | **10.3 ± 1.6** | **7.7 ± 2.0** | **8.1 ± 2.0** |

**Table S9:** Mean plasma HDL-cholesterol levels after treatment with NYX-PCSK9i, atorvastatin, or the combination of both. Numbers in **bold** are significantly different, compared to control.

|  | Plasma HDL-cholesterol (mmol/L) | | | |
| --- | --- | --- | --- | --- |
| Time (days) | 0 | 14 | 28 | 35 |
| Group 1: Vehicle control | 1.0 ± 0.1 | 1.1 ± 0.2 | 1.3 ± 0.1 | 0.9 ± 0.2 |
| Group 2: 50 mg/kg NYX-PCSK9i | 0.9 ± 0.1 | 0.7 ± 0.4 | **0.8 ± 0.2** | 0.7 ± 0.4 |
| Group 3: Atorvastatin control | 0.9 ± 0.2 | 1.1 ± 0.5 | 1.1 ± 0.5 | **0.5 ± 0.1** |
| Group 4: 50 mg/kg NYX-PCSK9i and atorvastatin | 0.9 ± 0.2 | **0.5 ± .1** | **0.6 ± 0.2** | 0.6 ± 0.1 |

**Table S10:** Mean plasma triglyceride levels were largely unchanged with NYX-PCSK9i, atorvastatin, or treatment with a combination of both. Numbers in **bold** are significantly different, compared to control.

|  | Plasma triglycerides (mmol/L; mean ± SD) | | | | | |
| --- | --- | --- | --- | --- | --- | --- |
| Time (days) | 0 | 7 | 14 | 21 | 28 | 35 |
| Group 1: Vehicle control | 5.3 ± 1.1 | 6.8 ± 2.3 | 4.9 ± 2.0 | 5.6 ± 3.2 | 3.4 ± 1.0 | 5.1 ± 2.0 |
| Group 2: 50 mg/kg NYX-PCSK9i | 5.3 ± 1.4 | 8.9 ± 3.3 | **8.6 ± 2.7** | 7.1 ± 1.6 | **6.0 ± 1.7** | 5.8 ± 1.7 |
| Group 3: Atorvastatin control | 5.6 ± 2.3 | 5.2 ± 2.0 | 5.9 ± 1.5 | 4.9 ± 1.7 | 5.1 ± 1.1 | 6.4 ± 1.6 |
| Group 4: 50 mg/kg NYX-PCSK9i and atorvastatin | 5.4 ± 2.2 | 7.2 ± 1.3 | 5.9 ± 1.1 | 4.7 ± 1.8 | 4.6 ± 1.4 | 3.7 ± 1.0 |

**Table S11:** No significant change in mean body weight of mice treated with NYX-PCSK9i, atorvastatin, or a combination of both was observed across 5 weeks of treatment

|  | Body weight (mean ± SD) | | | | | |
| --- | --- | --- | --- | --- | --- | --- |
| Time (days) | 0 | 7 | 14 | 21 | 28 | 35 |
| Group 1: Vehicle control | 22.4 ± 0.9 | 23.5 ± 1.2 | 23.4 ± 1.4 | 23.5 ± 1.2 | 23 ± 0.9 | 23.3 ± 1.2 |
| Group 2: 50 mg/kg NYX-PCSK9i | 21.9 ± 1.7 | 22.8 ± 2.0 | 23.0 ± 2.4 | 22.4 ± 2.0 | 21.9 ± 2.1 | 22.1 ± 1.9 |
| Group 3: Atorvastatin control | 21.7 ± 0.8 | 22.1 ± 0.8 | 22.3 ± 0.9 | 22.1 ± 0.9 | 21.8 ± 1.2 | 22.2 ± 1.3 |
| Group 4: 50 mg/kg NYX-PCSK9i and atorvastatin | 22.2 ± 1.5 | 22.7 ± 1.4 | 22.8 ± 1.8 | 22.6 ± 2.1 | 22.3 ± 2.0 | 22.1 ± 1.9 |

**Table S12:** No significant change in mean food intake of mice treated with NYX-PCSK9i, atorvastatin, or a combination of both was observed across 5 weeks of treatment

|  | Food intake (mean ± SD) | | | | | |
| --- | --- | --- | --- | --- | --- | --- |
| Time (days) | 0 | 7 | 14 | 21 | 28 | 35 |
| Group 1: Vehicle control | 3.4 ± 0.3 | 3.0 ± 0.0 | 2.8 ± 0.2 | 2.7 ± 0.1 | 2.4 ± 0.2 | 2.5 ± 0.3 |
| Group 2: 50 mg/kg NYX-PCSK9i | 3.4 ± 0.3 | 3.5 ± 0.3 | 3.4 ± 0.0 | 2.7 ± 0.0 | 2.7 ± 0.1 | 2.7 ± 0.3 |
| Group 3: Atorvastatin control | 3.4 ± 0.3 | 2.5 ± 0.1 | 2.5 ± 0.2 | 2.3 ± 0.0 | 2.3 ± 0.1 | 2.5 ± 0.1 |
| Group 4: 50 mg/kg NYX-PCSK9i and atorvastatin | 3.4 ± 0.3 | 2.8 ± 0.1 | 2.8 ± 0.4 | 2.4 ± 0.1 | 2.6 ± 0.1 | 2.5 ± 0.0 |

**Table S13:** No change in group-pooled levels of the liver enzyme ALT in mice treated with varying concentrations of NYX-PCSK9i was detected across 4 weeks of treatment

|  | Plasma ALT (U/L) | | | |
| --- | --- | --- | --- | --- |
| Time (days) | 0 | 14 | 28 | 35 |
| Group 1: Vehicle control | 98 | 69 | 46 | 56 |
| Group 2: 50 mg/kg NYX-PCSK9i | 91 | 84 | 36 | 42 |
| Group 3: Atorvastatin control | 101 | 90 | 49 | 57 |
| Group 4: 50 mg/kg NYX-PCSK9i and atorvastatin | 76 | 79 | 44 | 49 |

**Table S14:** No change in group pooled levels of the liver enzyme AST in mice treated with varying concentrations of NYX-PCSK9i was detected across 4 weeks of treatment

|  | Plasma AST (U/L) | | | |
| --- | --- | --- | --- | --- |
| Time (days) | 0 | 14 | 28 | 35 |
| Group 1: Vehicle control | 98 | 69 | 46 | 56 |
| Group 2: 50 mg/kg NYX-PCSK9i | 91 | 84 | 36 | 42 |
| Group 3: Atorvastatin control | 101 | 90 | 49 | 57 |
| Group 4: 50 mg/kg NYX-PCSK9i and atorvastatin | 76 | 79 | 44 | 49 |
